# Enhancing bulk defect-mediated absorption in silicon waveguides by doping compensation technique

**DOI:** 10.1038/s41598-018-28139-w

**Published:** 2018-07-02

**Authors:** Qiang Zhang, Hui Yu, Tian Qi, Zhilei Fu, Xiaoqing Jiang, Jianyi Yang

**Affiliations:** College of Information Science and Electronics Engineering, Zhejiang University, Hangzhou, 310027 China

## Abstract

Silicon waveguide photodiodes (SiWG PD) based on the bulk defect-mediated absorption (BDA) of sub-bandgap photons are suitable to realize in-line optical power monitors for silicon photonic integrated circuits. Deep-level states to enable the BDA can be induced by exploiting the ion implantation steps that are used to embed PN junctions for carrier-depletion-based modulators. This manner usually exhibits limited responsivities since relevant processing conditions are optimized for the modulation rather than the BDA. In this letter, we solve this issue with the doping compensation technique. This technique overlaps P-type and N-type implantation windows at the waveguide core. The responsivity is enhanced due to the increased density of lattice defects and the reduced density of free carriers in the compensated silicon. Influences of the dimension of the dopant compensation region on responsivity and operation speed are investigated. As the width of this region increases from 0 μm to 0.4 μm, the responsivity at −5 V is improved from 2 mA/W to 17.5 mA/W. This level is comparable to BDA based SiWG PDs relying on dedicated ion bombardments. On the other hand, a bit-error-rate test at 10 Gb/s suggests that the device with 0.2-μm-wide compensation region exhibits the highest sensitivity.

## Introduction

Application areas of silicon photonics technology gradually span from its initial motivation of optical interconnect to coherent optical communication, quantum computing^1^, sensing^2^, microwave photonics^3^, light detection and ranging (LIDAR)^4–6^, etc., during the last decade. In order to implement diverse advanced functions required by these emerging applications, both scale and complexity of photonic integrated circuits (PIC) on silicon-on-insulator (SOI) substrate grow rapidly. On the other hand, the strong optical confinement and the large thermo-optical coefficient of silicon waveguides make performances of silicon PICs very sensitive to fabrication tolerance and temperature fluctuation. Therefore, effective monitoring, control and stabilization of the operation point become indispensable for a large-scale silicon PIC. A solution to probe the beam propagation in a silicon waveguide is to tap a small fraction of the optical power to a Ge photodetector (PD) with a directional coupler. Although state-of-the-art Ge PDs grown on silicon possess high responsivities, this scheme is subjected to wavelength sensitivity of the directional coupler, limited scalability, and reliance on the high-quality growth of Ge layer. An alternative is to monitor the optical power in-line with a silicon waveguide PD (SiWG PD). Since the intrinsic absorption of silicon is negligible at wavelengths larger than 1.1 μm, SiWG PDs usually resort to two sub-bandgap absorption mechanisms, i.e., two-photon absorption (TPA) and defect-mediated absorption^7^. Photogenerated carriers then can be read out by pin/pn junctions^8–17^, metal-semiconductor-metal junction^18^, or photoconductor^19^. These conventional configurations all require direct contacts from electrodes to the waveguide core which result in additional optical losses. Therefore, a contactless integrated photonic probe is demonstrated to implement a non-invasive monitoring^20^.

As a nonlinear process, the TPA requires a high optical intensity to generate sufficient photocarriers. Also the quadratic dependence of the photocurrent on the optical power increases the control difficulty to some extent. In contrast, the linear defect-mediated absorption is better suited to monitor the optical power. Both bulk defects and surface states have associated deep energy levels in the bandgap, which allow sub-bandgap photons to excite electron-hole-pairs. The bulk defect-mediated absorption (BDA) usually relies on dedicated ion-implantation and annealing steps to induce lattice defects in silicon waveguides^12–18^. On the other hand, it is also observed that after the ion-implantations to embed the PN junction and the subsequent annealing to repair lattice damages, there are still residual lattice defects in silicon carrier-depletion-based modulators which provide a substantial photosensitivity to photons at C band^8–11^. This effect lends itself to monitor operation points of silicon PICs containing modulators very well since no additional processing steps are required.

The BDA is a two-step process that involves a photonic excitation of an electron from the valence band to the deep level and then a thermal excitation from the deep level to the conduction band, so its quantum efficiency is much lower than that of the intrinsic absorption. For example, responsivities of SiWG PDs by using residual defects in carrier-depletion-based modulators are usually on the order of several mA/W^8–11^. A weak photocurrent is adverse to build a simple, fast and robust feedback control loop. Although the responsivity can be boosted significantly by biasing the device in the avalanche regime, the high reverse bias voltage is ill-suitable for many applications.

In this paper, we demonstrate that the responsivity of the BDA is enhanced significantly in compensated silicon. The compensated silicon which contains both donor and acceptor species of similar doping concentrations is mainly used by solar cells in the past^21,22^. Low-cost solar-grade silicon materials usually contain high concentrations of unwanted dopants. Since purification steps to remove these impurities are rather expensive, an economical alternative to adjust the resistivity of silicon is to add compensating dopants. However, the compensated silicon usually exhibits degraded electrical properties including lifetime and mobility of carriers. It hence finds few applications in photonic or electrical integrated circuits. As will be demonstrated in the following context, the doping compensation technique presents an effective and economical solution to improve the quantum efficiency of the BDA based SiWG PD. This technique exploits the existing ion implantation steps that are used to make the silicon carrier-depletion-based modulator. The compensated silicon is realized by simply overlapping ion implantation windows of P-type and N-type dopants, so there is no additional processing cost. The improvement of the responsivity is thanks to two factors: Firstly, repeated ion implantations in the compensated silicon increase the density of lattice defects and consequently the sub-bandgap absorption^7^. Secondly, the compensation between donor and acceptor species decreases the density of free carriers inside the waveguide, so the free-carrier absorption which makes no contribution to the photocurrent is suppressed.

**Figure 1:**
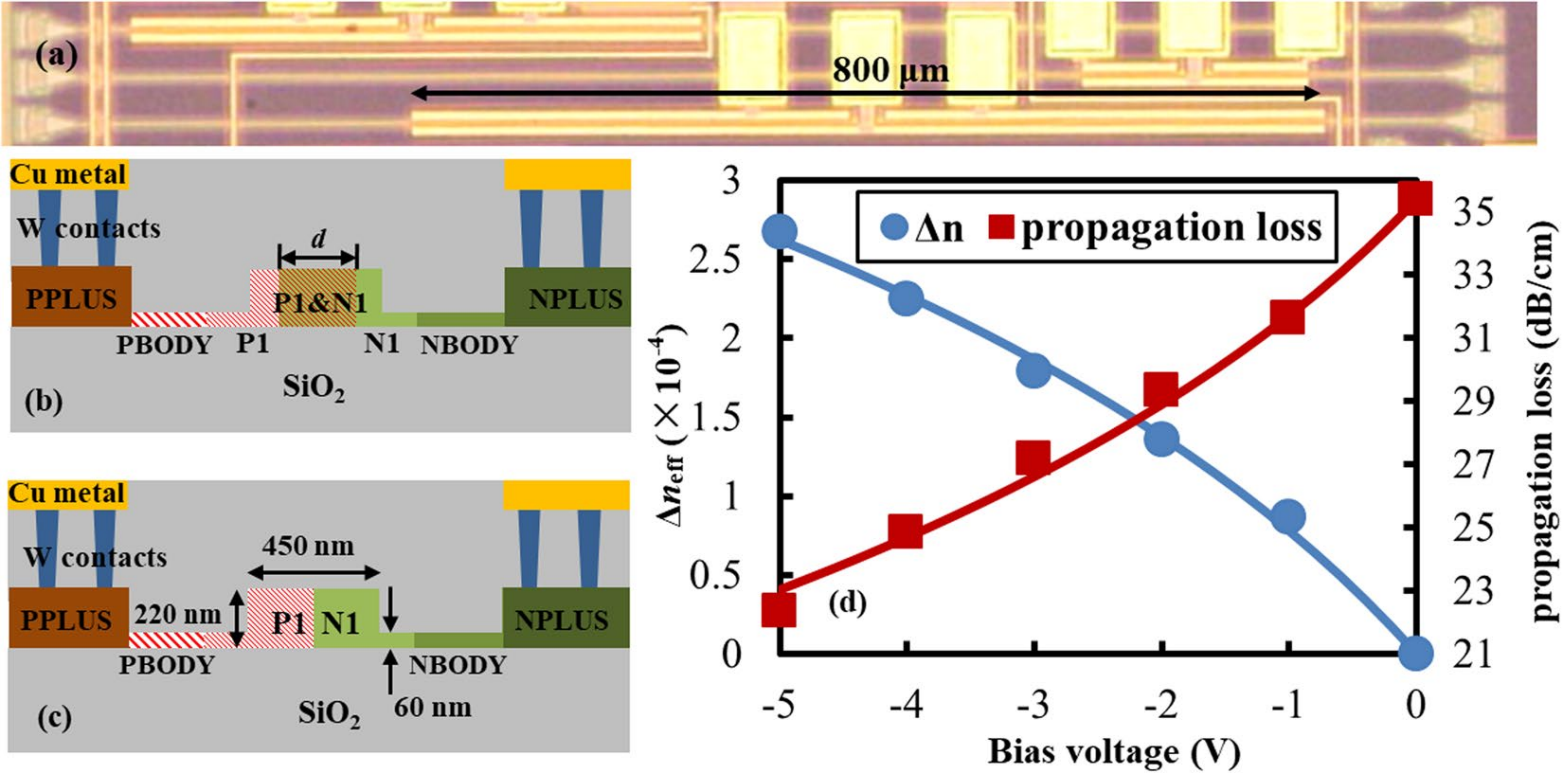
Micrograph, schematic cross-sections, and modulation characteristic of the devices. (**a**) Top-view microscope image of a SiWG PD based on compensated silicon. (**b**) Cross section and doping pattern of the same device. (**c**) Cross section and doping pattern of a carrier-depletion-based modulator on the same chip. (**d**) The modulation characteristic of the silicon modulator shown in (**c**).

The compensated silicon based SiWG PD is an ion-implanted straight rib waveguide as shown in Fig. 1(a,b). It is fabricated by the silicon photonics multi-project wafer (MPW) service of IMEC (ISIPP25G). The cross section of this device is very similar to the widely-used carrier-depletion-based silicon modulator shown in Fig. 1(c), except that the two doping windows to embed the PN junction overlap at the waveguide center. The SOI substrate has a buried oxide layer of 2 μm and a silicon layer of 220 nm. The width of the rib waveguide is 450 nm so as to accommodate only the fundamental TE mode. Increasing the etching depth on the one hand can enhance the lateral confinement to the optical mode, and therefore increases the optical intensity inside the carrier depletion region where the optical modulation occurs. This is favorable for improving the modulation efficiency. However, on the other hand, increasing the etching depth also leads to a high access resistance to the PN junction, and hence constrains the modulation bandwidth. An etching depth of 160 nm represents a reasonable trade-off between the efficiency and the bandwidth. As shown in Fig. 1(c), ISIPP25G contains six doping levels which are specifically optimized for the high-performance carrier-depletion-based modulator, i.e., P1/N1 to define the PN junction of the modulator, PBODY/NBODY to create low-resistivity electrical paths in silicon slabs, and PPLUS/NPLUS to create ohmic contacts. Since customers are not allowed to modify any ion implantation conditions of SIPP25G, no attempt is made to manipulate the compensation between the two dopants by engineering their implantation conditions. We set three different widths for the overlap between P1 and N1 implantation windows, which is *d* = 0/0.2/0.4 μm as shown in Fig. 1(b). A reference asymmetrical Mach-Zehnder (MZ) modulator is fabricated on the same chip as well. The waveguide cross-section of its phase shifter is shown in Fig. 1(c).

## Results

### Direct current (DC) performances of the devices

Transmission spectra of the reference MZ modulator under different reverse bias voltages are characterized at first. With the manner described in^23^, the effective index variation Δ*n*_eff_ and the absorption coefficient *α* of the guided mode at different reverse biases can be easily extracted from the measured spectra. The results are displayed in Fig. 1(d) as discrete points. The absorption coefficient *α* has been translated into the propagation loss in unit of dB/cm in Fig. 1(d). On the other hand, dependences of Δ*n*_eff_ and Δ*α* on the reverse bias *V*_b_ can be approximated by formulas Δ*n*_eff_ = *a·*ln(1 + *V*_b_/*V*_0_) and Δ*α* = *b·*ln(1 + *V*_b_/*V*_0_), respectively, where *a*, *b*, and *V*_0_ are constants which are determined by practical spatial distributions of dopants and the guided mode^24^. These two formulas then are used to fit the measured data. The fitting results are depicted in Fig. 1(d) as solid curves. The modulation efficiency is *V*_π_*L*_π_ = 1.7 V·cm as the reverse bias swings between 0 V and 5 V, which is in line with the specification provided by IMEC.

**Figure 2:**
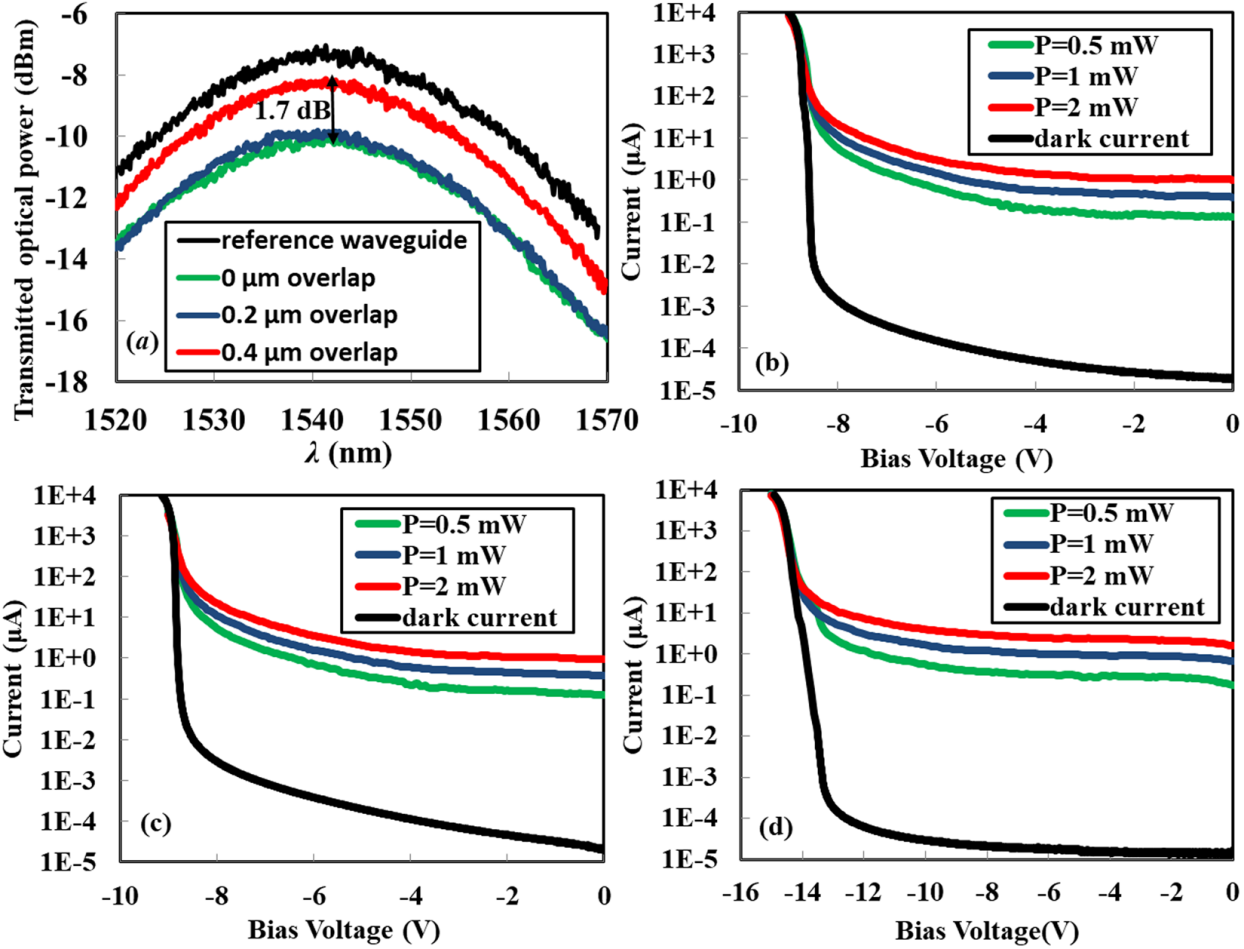
Transmission spectra and VI curves of the SiWG PDs. (**a**) Transmitted optical power versus the wavelength for compensated silicon based SiWG PDs with the width of the dopant compensation region as a variable. (**b**–**d**) VI curves of the same devices upon different on-chip incident optical powers. Widths of dopant compensation regions are *d* = 0/0.2/0.4 μm for (b)/(c)/(d). The on-chip optical power incident on the SiWG PD is estimated by subtracting the loss of the fiber grating coupler from the output power of the laser.

Transmitted optical powers through 800-μm-long SiWG PDs are plotted in Fig. 2(a) as a function of the wavelength. In order to remove the influence of the two fiber grating couplers, the transmitted power of a straight strip waveguide of the same length is plotted as a reference. According to the data in Fig. 2(a), propagation losses of compensated silicon based rib waveguides are 36/34/14 dB/cm when *d* = 0/0.2/0.4 μm. We note that for the propagation loss of the doped waveguide with *d* = 0 μm, the results shown in Figs 1(d) and 2(a) coincide with each other. The sidewall roughness of silicon rib waveguides due to the etching usually induces a scattering loss of ~2 dB/cm^25^. The propagation losses we measured in Fig. 2(a) thus are dominated by the optical absorption of free carriers in doped waveguides. As the overlap rises from 0 μm and 0.4 μm, the propagation loss falls dramatically. It implies that the compensation between the two dopants effectively reduces the density of free carriers which cause a strong optical absorption. However, it is also found that the transmitted power of the SiWG PD with a 0.2-μm-wide overlap is only slightly stronger than that with no overlap. We attribute this to the lateral straggling of implanted ions, and the inevitable alignment error of lithography which is on the scale of several tens of nanometers^26^. These two factors lead to a considerable compensation even if the two masks to define N1/P1 implantation windows have no overlap. As a result, it is possible that the net doping concentrations at the waveguide center are close for *d* = 0 μm and *d* = 0.2 μm. The VI characteristics of the three devices shown in Fig. 2(b,d) further manifest the compensation between dopants. As we expected, the two devices with *d* = 0/0.2 μm exhibit very close breakdown voltages of around −8.5 V, while the third device of *d* = 0.4 μm which has the lowest net doping concentration presents the highest breakdown voltage of around −13.5 V.

In the low bias region where the carrier multiplication mechanism plays no role, the SiWG PD with *d* = 0.4 μm produces the highest primary photocurrent. Specifically, photocurrents in Fig. 2(d) are around 2~3 times as high as those in Fig. 2(b) upon the same bias condition and the same incident optical power. We note that incident optical powers are not completely absorbed by the three SiWG PDs due to their limited length of 800 μm. Since the device with 0.4-μm-wide compensation region has the lowest propagation loss and thus absorbs the fewest optical power, we expect that the enhancement factor of the internal responsivity is higher than that of the measured primary photocurrent.

**Figure 3:**
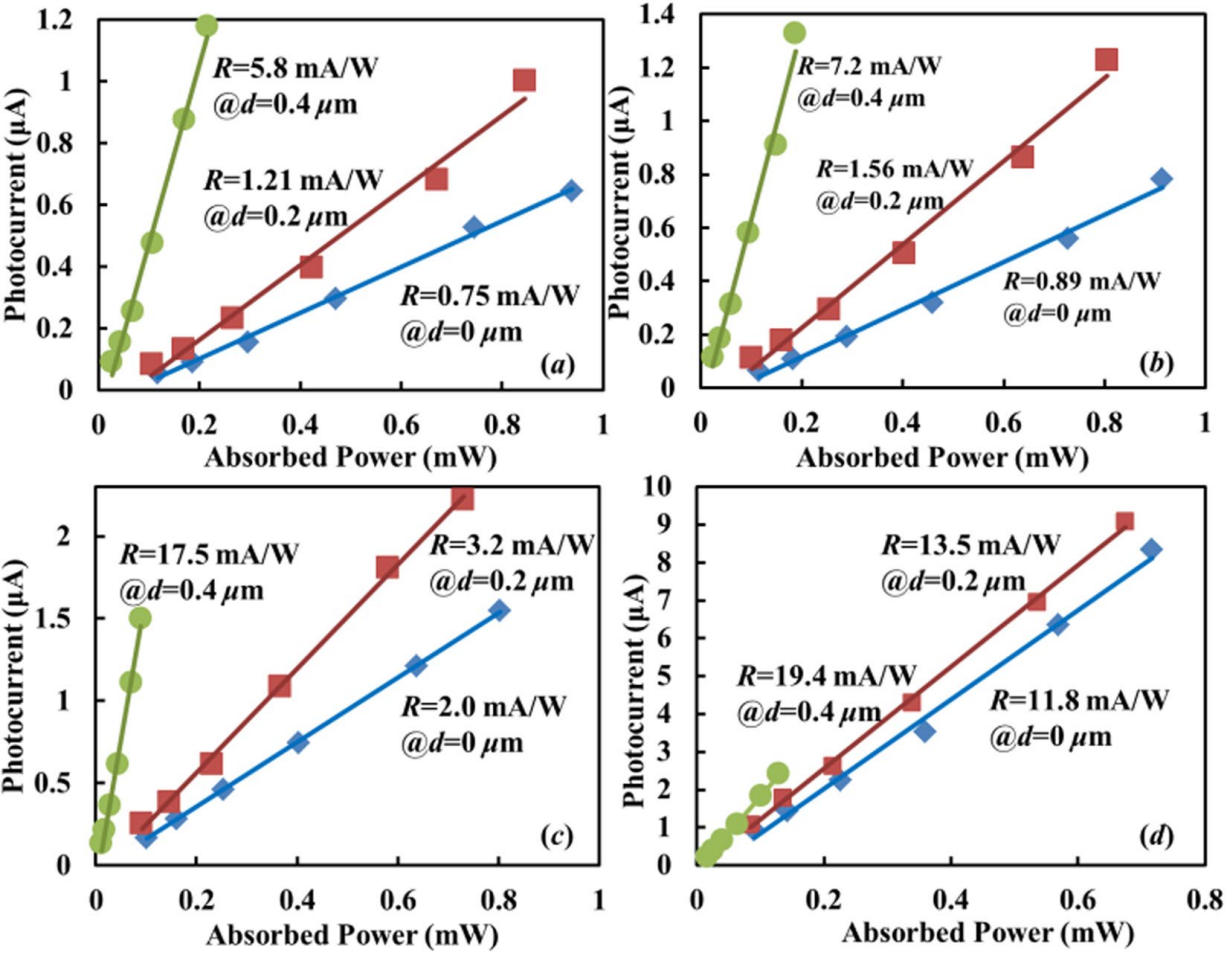
Responsivities of the SiWG PDs at different biases. Photocurrent versus net absorbed optical power for the three devices under different bias voltages of (**a**) −1 V, **(b**) −3 V, (**c**) −5 V, (**d**) −7 V. Discrete points are the measured data. Solid curves are the linear fitting results. Their slopes represent internal responsivities.

Measured internal responsivities are plotted in Fig. 3 for the three devices under different reverse bias voltages. The internal responsivity *R* is defined as the photocurrent divided by the net absorbed optical power, i.e., the on-chip optical power entering the device minus that exiting the device. As shown in Fig. 3(a–c), the device with *d* = 0.4 μm exhibits an around ×8 improvement of the internal responsivity upon *V*_b_ = −1/−3/−5 V. This improvement factor drops to be less than two in Fig. 3(d) where the reverse bias is −7 V. The reason is that under this bias condition, the carrier multiplication effect starts to play a role when *d* = 0/0.2 μm, while it is still insignificant when *d* = 0.4 μm.

### The noise-equivalent powers (NEPs) and the temperature dependences of responsivities

**Figure 4:**
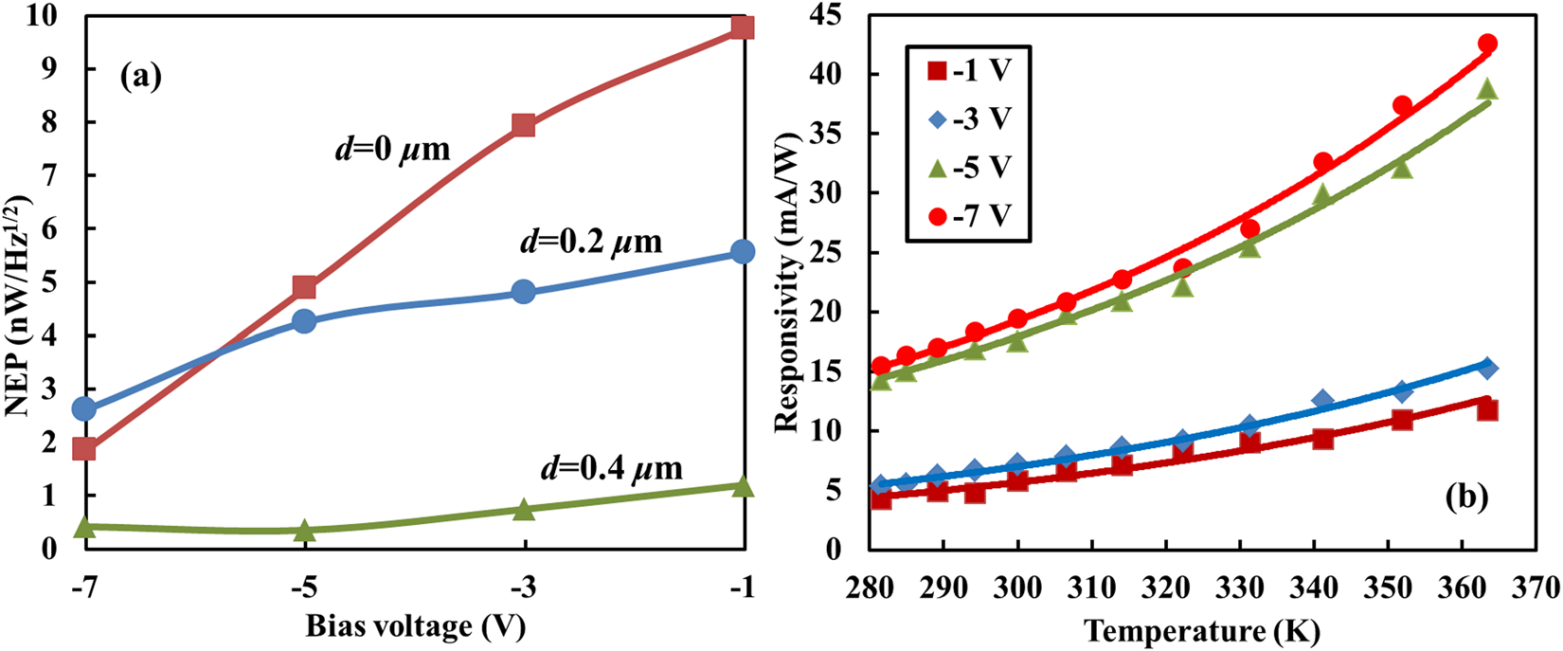
NEP and temperature dependence of responsivities. (**a**) NEPs of the three SiWG PDs at different biases. (**b**) Responsivity of the SiWG PD with *d* = 0.4 μm versus temperature.

Based on the responsivities measured in Fig. 3, we experimentally determine noise-equivalent powers (NEPs) of the three devices under different biases, and then plot the result in Fig. 4(a). The procedure to quantify the NEPs is introduced in Methods. As expected, the SiWG PD with *d* = 0.4 μm always exhibits the lowest NEP among the three devices thanks to its high responsivity. We also note that the magnitude of NEPs shown in Fig. 4(a) coincides with those measured in^17^.

The temperature dependence of the responsivity is also characterized and then plotted in Fig. 4(b) for the device with *d* = 0.4 μm. Discrete points denote responsivities measured at different temperatures and bias voltages. Solid curves represent exponential functions used to fit the measured data. It can be seen that they capture dependences of responsivities on temperature quite well. Regardless of bias voltages, responsivities are enhanced by a factor of ~2.1 as the temperature rises from 300 K to 365 K. Such an exponential dependence can be attributed to the thermal excitation step involved in the BDA. According to the Shockley-Read-Hall (SRH) generation/recombination theory, the rate of electrons to be promoted from the deep-level states associated with lattice defects to the conduction band increases with the temperature^7,27^.

### The bit-error-rate measurement

In most feedback-control loops of silicon PICs, power monitors are used to probe variations of average optical powers against low-frequency dithering signals or temperature fluctuations, so a high detection speed is not demanded. However, there are also application scenarios which measure bit error rates (BER) directly as feedback signals, such as wavelength locking and thermal stabilization of silicon ring modulators^28^. On the other hand, BDA based SiWG PDs hold the potential for high speed optical communications in the mid-infrared region^17^. Therefore, it is also necessary to investigate the influence of the compensated silicon on the operation speed.

**Figure 5:**
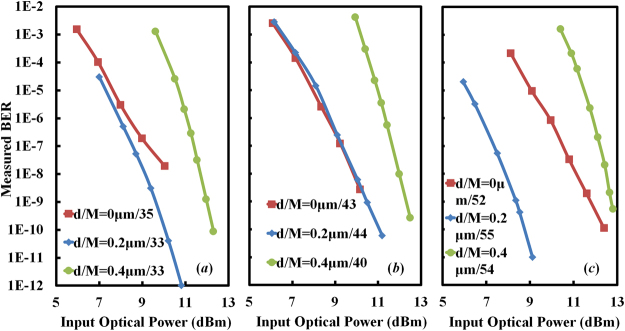
Bit-error-rate versus on-chip optical power of the three devices upon different multiplication factors. The non-return-to-zero (NRZ) pseudo-random binary sequence (PRBS) signal used to modulate the input optical carrier has a data rate of 10 Gb/s and a word length of 2^31^-1.

Dynamic responses of the three devices are characterized by measuring their eye diagrams and BERs. The three devices operate in the avalanche regime so as to generate sufficient photocurrents. During the high speed measurement, demodulated current signals are directly sent to a BER tester or a sampling oscilloscope without using a trans-impedance amplifier. The procedure of high-speed measurement is similar to that in^16^. Details of the high-speed measurement setup are provided in Methods. Figure 5 displays measured sensitivity curves of the three devices under different multiplication factors *M*. The multiplication factor is defined as the ratio of the multiplied photocurrent to the primary photocurrent. We assume that the value of *M* is independent of the input optical power and the unit gain occurs at −3V. Since the bandwidth of an avalanche PD relies on the multiplication factor, sensitivities of the three devices are compared in Fig. 5 when their multiplication factors are close. We find that although the device with 0.4-μm-wide compensation region outperforms the other two devices in terms of the insertion loss and the DC responsivity, it is subjected to the lowest sensitivity. We attribute this disadvantage to its limited gain-bandwidth product.

It is well known that the bandwidth of an avalanche PD is constrained by two factors, i.e., the avalanche built-up time that is characterized by the gain-bandwidth product, and the RC time constant. Junction capacitances of the three devices measured near their breakdown voltages are 200/200/150 fF when *d* = 0/0.2/0.4 μm. According to the formula *f*_RC_ = 1/2π*RC*, we estimate that RC limited bandwidths of the three devices are 15.9/15.9/21.2 GHz by assuming a total series resistance of 50 Ω. Therefore, RC time constants of all three devices are short enough to support the detection at 10 Gb/s. On the other hand, an empirical formula to estimate the gain-bandwidth product is *M*·*B* = 1/[2π*N*(*α*_p_/*α*_n_)*τ*_av_]^29^. Here, *α*_p_ and *α*_n_ denote impact ionization coefficients of holes and electrons, respectively. *N* is the slow-varying function of the ratio *α*_p_/*α*_n_. Its value is between 1/3 and 2 as the value of *α*_p_/*α*_n_ varies between 1 and 0.001. The average transit time *τ*_av_ is determined by the width of the carrier depletion region *w*, the electron saturation velocity *v*_n_, and the hole saturation velocity *v*_p_ as *τ*_av_ = (*w*/*v*_n_ + *w*/*v*_p_)/2^29^. As an approximation, we assume the net doping concentration in the dopant compensation region is zero. Therefore, if *d* = 0.4 μm, the ~14 V avalanche voltage leads to an electrical field strength of ~3.5 × 10^5^ V/cm in the “intrinsic” dopant compensation region. D. J. Massey has summarized dependences of impact ionization coefficients on electrical field strength and temperature for silicon pin diodes whose intrinsic regions are in sub-micrometer scale^30^. His result suggests that the ionization coefficient ratio *α*_p_/*α*_n_ is ~0.11 for the specific electric field level here. The corresponding value of *N* then is *N* = 1^29^. Substituting *w* ≈ *d* = 0.4 μm, *v*_n_ = *v*_p_ = 10^7^ cm/s, *N* = 1, *α*_p_/*α*_n_ = 0.11 into the formula of *M*·*B* yields a gain-bandwidth product of < 360 GHz. The multiplication factor of *M* = 54 in Fig. 5(c) thus implies a bandwidth of no more than 6.7 GHz, which is marginal for the detection at 10 Gb/s. In contrast, the device with *d* = 0.2 μm is expected to have a higher gain-bandwidth product due to the reduced width of the dopant compensation region, so it presents the best sensitivity in Fig. 5.

### The eye diagram measurement

**Figure 6:**
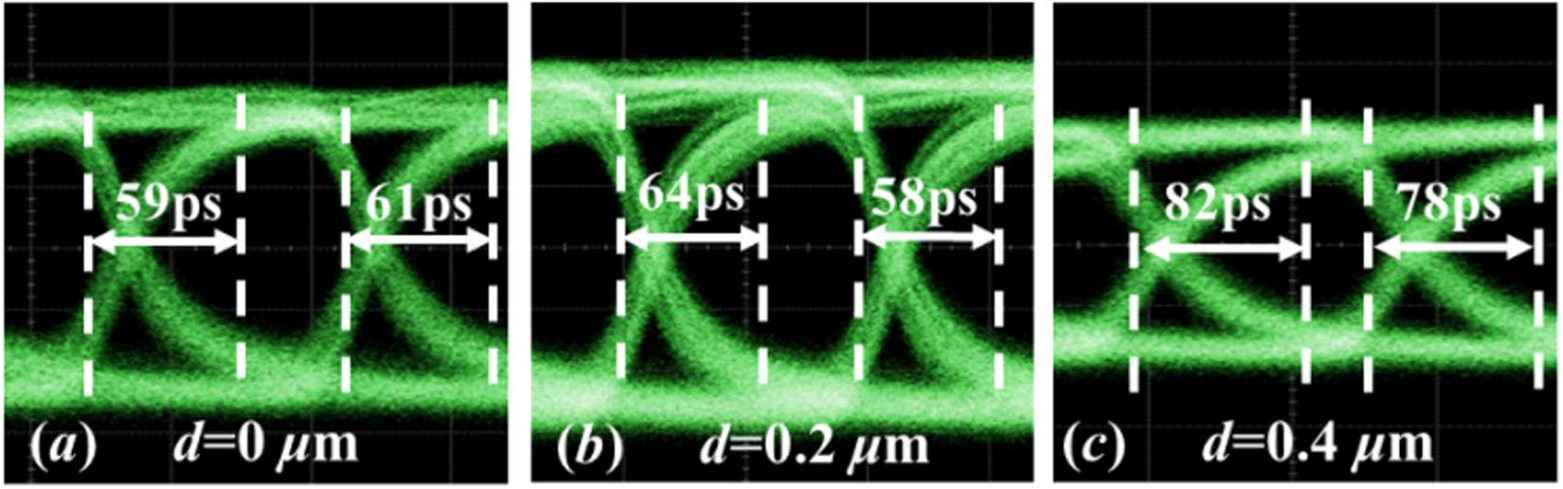
Eye diagrams of the three devices in the avalanche mode at a data rate of 10 Gb/s.

Different response speeds of the three devices are also manifested by their eye diagrams in Fig. 6, which are measured at a bit rate of 10 Gb/s when their multiplication factors are around *M* = 40. The 10–90% rise times and the 90%−10% falling times read out by the oscilloscope are marked inside the eyes. It is apparent that the device with *d* = 0.4 μm exhibits the longest rising and falling edges. From left to right, measured signal to noise ratios are 6.9, 7.1 and 5.6. These results indicate that although a wide compensation region is favorable to enhance the responsivity in DC, it is adverse to the operation speed in the avalanche mode. We suggest that the value of *d* should be chosen according to specific requirements of speed and responsivity.

## Discussion

To further demonstrate the advantage of the doping compensation technique, our device with 0.4-μm-wide compensation region is compared favorably with reported BDA based SiWG PDs in Table 1.

**Table 1:** A comparison of the DC responsivity between our device and previously reported BDA based SiWG PDs at a wavelength of 1550 nm.

Device	Type	Defect formation	*R* (mA/W)
X. Li^9^	microring/PN diode	Modulator	2.3/5.1 @ −3/−5 V
Y. Li^10^	microring/PN diode	Modulator	2.2/3.6 @−3/−5 V
Z. Wang^11^	microring/PN diode	Modulator	0.18 @−4V
D. J. Thomson^12^	straight wg/PIN diode	4 MeV B^+^ implantation	6~10
J. J. Ackert^13^	straight wg/PIN diode	500 keV B^+^ implantation	2.2/3.2 @−3/−5 V
B. Souhan^18^	straight wg/MSM diode	195 keV Si^+^ implantation	10.5 @−5 V
D. F. Logan^14^	Straight wg/PIN diode	350 keV Si^+^ implantation	43 @−5 V
D. F. Logan^15^	Straight wg/PIN diode	190 keV Si^+^ implantation	5.1/40 @−5/−20 V
R. R. Grote^16^	Straight wg/PIN diode	Si^+^ implantation	12/22 @−3/−5 V
Our Device *d* = 0.4 μm	Straight wg/PN diode	doping compensation	7.2/17.5 @−3/−5 V

We note that many of reported devices utilize optical cavities to enhance the optical power inside the waveguide, while the others are based on straight waveguides which absorb different fractions of input lights. Definitions of the responsivity are not uniform in these reports. In order to make a fair comparison, we calculate internal responsivities *R* of these devices, i.e., the ratio between the photocurrent to the net absorbed optical power rather than the incident optical power, according to the data available in these reports. An exception is the device reported by R. R. Grote in the tenth row. The insertion loss of this device necessary for calculating the internal responsivity is not given in^9^, so its external responsivity is listed instead. The term “modulator” in the third column implies that the device in this row is initially designed as a carrier-depletion-based modulator.

The comparison indicates that internal responsivities of BDA based SiWG PDs are on the scale of several mA/W by directly leveraging residual lattice defects in silicon carrier-depletion-based modulators, while dedicated ion bombardment and annealing steps are able to enhance internal responsivities to a level of several tens of mA/W. The doping compensation technique offers an internal responsivity that is comparable or even better than those achieved by dedicated ion implantation and annealing steps.

Despite we are not allowed to adjust the doping condition of MPW service, it is still advisable to discuss the influence of the doping concentration. Since the BDA based SiWG PD we proposed here shares the same ion implantation steps used to make the silicon modulator, the performance of the modulator is of paramount importance if we are free to choose the doping concentration. Influence of the doping concentration on the silicon modulator has been investigated quite extensively in the past^23,26^. According to the related theory, the modulation efficiency of a silicon modulator is proportional to the square root of the doping concentration. However, increasing the doping concentration also leads to a strong optical absorption of free carriers as well as a large capacitance of the PN junction. Based on a trade-off between efficiency, insertion loss and bandwidth, most silicon modulators have their doping concentrations in the range from 2 × 10^17^/cm^3^ to 1 × 10^18^/cm^3^^23,31^. On the other hand, the responsivity of the BDA depends on the defect concentration as a result of two conflicting mechanisms. Firstly, the rate of electrons to be excited from the valence band to empty defect states by sub-bandgap photons is proportional to the defect concentration, so increasing the defect concentration can enhance the BDA. Secondly, defect states also act as recombination centers which would reduce the collection efficiency of photon-generated carriers. A model based on modified SRH theory suggests that there is an optimal defect concentration to produce the highest responsivity^27^. During the ion implantation, the concentration of defects should be proportional to that of the doping. Based on these arguments, we believe that an optimal doping concentration can be found by taking into account the BDA based SiWG PD and the modulator together.

In summary, we demonstrate that the responsivity of the BDA based SiWG PD can be substantially enhanced by the dopant compensation technique. The technique is completely based on the regular doping process used to realize the carrier-depletion-based modulator. It is therefore particularly suitable for monitoring silicon PICs which contain the functionality of modulation. We want to stress that the improvement of the internal responsivity depends on how complete the compensation is, i.e., the net doping concentration in the dopant compensation region. A highly compensated silicon waveguide requires a fine control of doping profiles by engineering related doping processes^26^. However, the ion implantation conditions of the current MPW service are optimized only for the performance of the modulator. We believe that if relevant processing conditions can be optimized globally by considering performances of both carrier-depletion-based modulator and compensated silicon based SiWG PD, the responsivity can be further improved.

## Methods

### NEP measurement

The procedure to measure the NEP is similar to that described in^32^. A semiconductor device parameter tester (Keysight B1500A) is used to sample the dark current of the device with a certain sampling frequency. The measured sequence of dark currents then is used to calculate the power spectral density *S* of the dark-current noise via the Fourier transform. According to its definition, the NEP can be calculated as NEP = (*S*)^1/2^/*R*, where *R* is the responsivity of the SiWG PD under test.

### High-speed measurement setup

**Figure 7:**
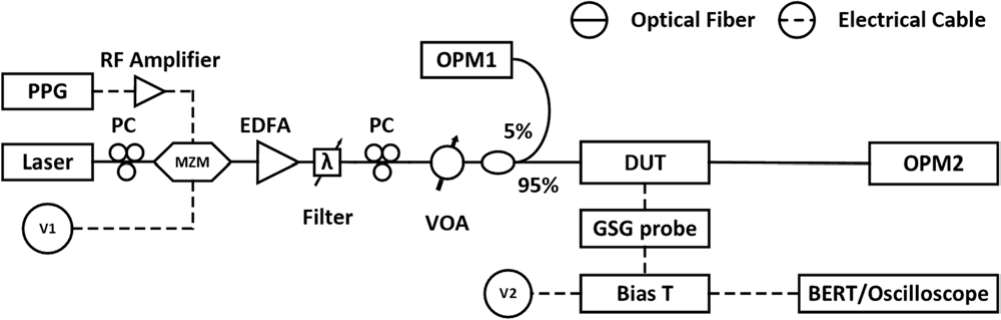
Experimental setup to measure the BER and the eye diagram.

Our experimental setup to measure BER and eye diagram is shown in Fig. 7. A tunable laser (Santec TSL-510) operating at 1550 nm is connected to a 12 GHz LiNbO_3_ modulator (Photline MX-LN-10) biased at the quadrature point through a polarization controller (PC). A pulse-pattern generator (PPG) (Keysight N4951B) produces a 2^31^-1 pseudo-random binary sequence (PRBS) non-return-to-zero (NRZ) signal at 10 Gb/s. The signal is amplified by a radiofrequency (RF) amplifier before driving the LiNbO_3_ modulator. The output of the modulator is sent to an erbium-doped fiber amplifier (EDFA) (Keopsys CPB33) which is followed by an optical filter to reduce the amplified spontaneous emission noise and a variable optical attenuator (VOA) to adjust the optical power level in the link. Two fiber grating couplers serve as interfaces between input/output ports of the SiWG PD and single mode fibers. Another PC before the DUT ensures that the optical field incident on the fiber grating coupler is TE polarized. In order to monitor the average optical power being launched on chip from the fiber, a tap couples 5% of the incident light to an optical power meter (OPM) (HP 8153 A). The on-chip optical power entering the SiWG PD is estimated by subtracting the coupling loss of the fiber grating coupler from the incident optical power. Another optical power meter is used to monitor the optical power exiting the chip. The net absorbed power is calculated as the difference between the optical powers entering and exiting the SiWG PD.

The electrical contact is implemented by a 40 GHz GSG probe. A bias tee is used to decouple the direct current (DC) and the alternating current (AC) components of the measured photocurrent. The DC component is measured by a sourcemeter (Keithley 2400) which provides the reverse bias voltage. The AC component is directly sent to a bit-error-rate (BER) tester (Keysight 4952 A) for the BER measurement, or to a sampling oscilloscope (Keysight 86105D) for the eye diagram measurement. There is no transimpedance amplifier (TIA) in the measurement setup.

## References

[1] Silverstone JW, Bonneau D, Silverstone JW, Thompson MG (2016). silicon quantum photonics. IEEE J. Sel. Top. Quantum Electron..

[2] Gavela AF, García DG, Ramirez JC, Lechuga LM (2016). Last advances in silicon-based optical biosensors. Sensors.

[3] Pérez D (2017). Multipurpose silicon photonics signal processor core. Nat. Commun..

[4] Hutchison DN (2016). High-resolution aliasing-free optical beam steering. Optica.

[5] Abediasl H, Hashemi H (2015). Monolithic optical phased-array transceiver in a standard SOI CMOS process. Opt. Express.

[6] Poulton CV (2017). Coherent solid-state LIDAR with silicon photonic optical phased arrays. Opt. Lett..

[7] Casalino M, Coppola G, De La Rue RM, Logan DF (2016). State-of-the-art all-silicon sub-bandgap photodetectors at telecom and datacom wavelengths. Laser Photonics Rev..

[8] Yu H (2012). Using carrier-depletion silicon modulators for optical power monitoring. Opt. Lett..

[9] Li X (2015). 40 Gb/s all-silicon photodetector based on microring resonators. IEEE Photon. Technol. Lett..

[10] Li Y, Feng S, Zhang Y, Poon AW (2013). Sub-bandgap linear-absorption-based photodetectors in avalanche mode in PN-diode-integrated silicon microring resonators. Opt. Lett..

[11] Wang Z (2017). Resonance control of a silicon micro-ring resonator modulator under high-speed operation using the intrinsic defect-mediated photocurrent. Opt. Express.

[12] Thomson DJ (2014). Optical detection and modulation at 2µm-2.5µm in silicon. Opt. Express.

[13] Ackert JJ (2013). 10 Gbps silicon waveguide-integrated infrared avalanche photodiode. Opt. Express.

[14] Logan DF (2012). Monitoring and Tuning Micro-Ring Properties Using Defect-Enhanced Silicon Photodiodes at 1550 nm. IEEE Photon. Technol. Lett..

[15] Logan DF (2010). Defect-Enhanced Silicon-on-Insulator Waveguide at 1.55 μm. IEEE Photon. Technol. Lett..

[16] Grote RR (2013). 10 Gb/s error-free operation of all-silicon ion-implanted-waveguide photodiodes at 1.55 μm. IEEE Photon. Technol. Lett..

[17] Ackert JJ (2015). High-speed detection at two micrometres with monolithic silicon photodiodes. Nat. Photonics.

[18] Souhan B (2014). Si^+^-implanted Si-wire waveguide photodetectors for the mid-infrared. Opt. Express.

[19] Jayatilleka H (2015). Wavelength tuning and stabilization of microring-based filters using silicon in-resonator photoconductive heaters. Opt. Express.

[20] Grillanda S (2014). Non-invasive monitoring and control in silicon photonics by CMOS integrated electronics. Optica.

[21] Dubois S, Enjalbert N, Garandet JP (2008). Effects of the compensation level on the carrier lifetime of crystalline silicon. Appl. Phys. Lett..

[22] Macdonald D, Cuevas A (2011). Recombination in compensated crystalline silicon for solar cells. J. Appl. Phys..

[23] Yu H (2012). Performance tradeoff between lateral and interdigitated doping patterns for high speed carrier-depletion based silicon modulators. Opt. Express.

[24] Vacondio F (2010). A Silicon Modulator Enabling RF Over Fiber for 802.11 OFDM Signals. IEEE J. Sel. Top. Quantum Electron..

[25] Bogaerts W (2010). Silicon-on-insulator spectral filters fabricated with CMOS technology. IEEE J. Sel. Top. Quantum Electron..

[26] Yu H, Bogaerts W, De Keersgieter A (2010). Optimization of ion implantation condition for depletion-type silicon optical modulators. IEEE J. Quantum Electron..

[27] Logan DF, Jessop PE, Knights AP (2009). Modeling Defect Enhanced Detection at 1550 nm in Integrated Silicon Waveguide Photodetectors. J. Light. Technol..

[28] Zortman WA, Lentine AL, Trotter DC, Watts MR (2013). Bit-error-rate monitoring for active wavelength control of resonant modulators. IEEE Micro.

[29] Sze, S. M. & Ng, K. K. *Physics of Semiconductor Devices* (Wiley, 2007).

[30] Massey DJ, David JPR, Rees GJ (2006). Temperature dependence of impact ionization in submicrometer silicon devices. IEEE Trans. Electron Devices.

[31] Li M, Wang L, Li X, Xiao X, Yu S (2018). Silicon intensity Mach–Zehnder modulator for single lane 100  Gb/s applications. Photonics Res..

[32] An X, Liu F, Jung YJ, Kar S (2013). Tunable graphene-silicon heterojunctions for ultrasensitive photodetection. Nano Lett..

